# Therapeutical Notes

**Published:** 1900-02-17

**Authors:** 


### THERAPEUTICAL NOTES.

### SHINGLES.

DELEBECQUE (*Thèse de Paris*) recommends the application of picric acid, dissolved in water, alcohol, or ether. The two latter are more painful at the moment, but are preferable, especially for exposed surfaces. The aqueous solution, 12 parts in 1,000, is the same as that used for superficial burns. Absorbent compresses are wrung out in the solution and bandaged over the affected area, but no protective or other impermeable covering is employed. The dressing is removed very gently and changed every three or four days. The acid has valuable antiseptic properties, and allays in a marked manner the pain and itching.—*Merck's Archives*, December, 1899.

